# RNA-Seq comparative study reveals molecular effectors linked to the resistance of *Pinna nobilis* to *Haplosporidium pinnae* parasite

**DOI:** 10.1038/s41598-022-25555-x

**Published:** 2022-12-08

**Authors:** Pauline Salis, Claire Peyran, Titouan Morage, Simon de Bernard, Julien Nourikyan, Stéphane Coupé, Robert Bunet, Serge Planes

**Affiliations:** 1 PSL Research University: EPHE-UPVD-CNRS, UAR 3278 CRIOBE, 66860 Perpignan, France; 2 AltraBio, 69007 Lyon, France; 3 CNRS/INSU, IRD, MIO UM 110, Mediterranean Institute of Oceanography, University of Toulon, 83130 La Garde, France; 4 Institut Océanographique Paul Ricard, Ile des Embiez, 83140 Six-Fours-Les-Plages, France; 5 Laboratoire d’Excellence “CORAIL”, Perpignan, France

**Keywords:** Ecology, Genetics, Immunology

## Abstract

With the intensification of maritime traffic, recently emerged infectious diseases have become major drivers in the decline and extinction of species. Since 2016, mass mortality events have decimated the endemic Mediterranean Sea bivalve *Pinna nobilis,* affecting ca. 100% of individuals. These events have largely been driven by *Haplosporidium pinnae*’s infection, an invasive species which was likely introduced by shipping. While monitoring wild populations of *P. nobilis*, we observed individuals that survived such a mass mortality event during the summer of 2018 (France). We considered these individuals resistant, as they did not show any symptoms of the disease, while the rest of the population in the area was devastated. Furthermore, the parasite was not detected when we conducted a PCR amplification of a species-specific fragment of the small subunit ribosomal DNA. In parallel, the transcriptomic analysis showed evidence of some parasite RNA indicating that the resistant individuals had been exposed to the parasite without proliferating. To understand the underlying mechanisms of resistance in these individuals, we compared their gene expression with that of susceptible individuals. We performed de novo transcriptome assembly and annotated the expressed genes. A comparison of the transcriptomes in resistant and susceptible individuals highlighted a gene expression signature of the resistant phenotype. We found significant differential expressions of genes involved in immunity and cell architecture. This data provides the first insights into how individuals escape the pathogenicity associated with infection.

## Introduction

Over the past decades, an increase in the prevalence and severity of emerging infectious diseases that affect wild populations has been observed, often linked to global warming^1–3^, and mostly leading to dramatic population losses^3–6^. One of the most frequently cited cases is the increase in malaria infection found in wild birds, which has been linked to a warming climate^7,8^. In marine biota, multiple diseases (white plague disease and ciliate infections) have impacted Caribbean coral (*Diploria labyrinthiformis*) during the 2010 warm thermal anomaly in Curaçao^9,10^. However, due to the lack of long-term records, it is difficult to evaluate how global warming and anthropogenic drivers affect the emergence and intensity of infectious diseases and how they may affect marine biodiversity and the survival of the species^11^. Several authors have suggested that the Mediterranean Sea could be used as a natural laboratory. This could serve as a miniature model of the world’s oceans to help study this question^12^.

Sentinel species such as the Mediterranean endemic fan mussel *Pinna nobilis*^13^ could provide information on what the future holds for global ecology within the current context of global warming. Since early autumn 2016, this bivalve has been affected by mass mortality events caused by an emerging protozoan parasite, *Haplosporidium pinnae*^14^, which has decimated *P. nobilis* throughout its entire distribution range^15^. For nearly 2 years, mass mortalities have affected fan mussel populations throughout all Mediterranean coasts^16^. It is therefore reasonable to suggest that the endangered *P. nobilis* could be facing total extinction over the medium, or even short-term time scales^16^. In addition to the pathogenicity of the parasite^14^, previous model simulations indicate that an increase in water temperature is related to the proliferation of the disease with peak mortality in the summer season, which is strongly influenced by climate change and anthropogenic actions^16–18^.

While most studies^17,18^ have focused on understanding the factors that condition the spread of the die-off (such as population dynamic surveys^19^) and the implementation of protection programs (larvae collectors and protection of infected adults from predators^16^), very few have focused on transmission dynamics or the etiology of the disease such as the molecular mechanisms of pathogenicity of the emerging parasite^14,20^. From now, histological studies have identified that the parasite causes a strong inflammatory response, linked to considerable infiltration of the digestive glands by the parasite, which likely blocks the digestive system and thus leads to the starvation, severe general dysfunction, and death of the host^14,20^. Moreover, the bivalve stops being able to produce an antioxidant response^20^. To better understand the capacity of the host to survive the parasite means finding individuals that survive the infection.

Existence of *P. nobilis* survivors after an *H. pinnae* infection is very rare^17^. To date, no study has examined the molecular mechanisms that may have enabled these individuals that were likely exposed to the virulent pathogen to survive. In this study, we report four live individuals that were identified in 2019, after an intensive monitoring effort, in an area in which the *P. nobilis* population was decimated by an *H. pinnae* infection during summer 2018 (French coast, Peyrefite bay, Marine Protected Area of Cerbère-Banyuls, France)^21^.

First, we verified whether we could detect the parasite in the tissue of the resistant individuals. While we were unable to detect the parasite using PCR amplification of a species-specific fragment of the SSU rDNA, the transcriptomic analysis showed evidence of some parasite RNA in the mantle tissue, demonstrating that while the individuals were exposed to the parasite, the parasite did not proliferate.

To understand this resistance to *H. pinnae*, we performed a comparative transcriptomic analysis between *P. nobilis* individuals susceptible and resistant to *H. pinnae*. Through principal component analyses (PCA) and hierarchical analysis, we researched the formation of clusters of resistant and susceptible individuals. Interestingly, the PCA showed a cluster of resistant and another of susceptible and highlighted a different transcriptional signature between these two groups of individuals^22,23^. Moreover, we observed that in resistant *P. nobilis*, the transcriptional signature is associated with biological processes linked to immunity and cell architecture.

## Results

### Live *P. nobilis* individuals are found in an infected zone of *H. pinnae*

**Figure 1:**
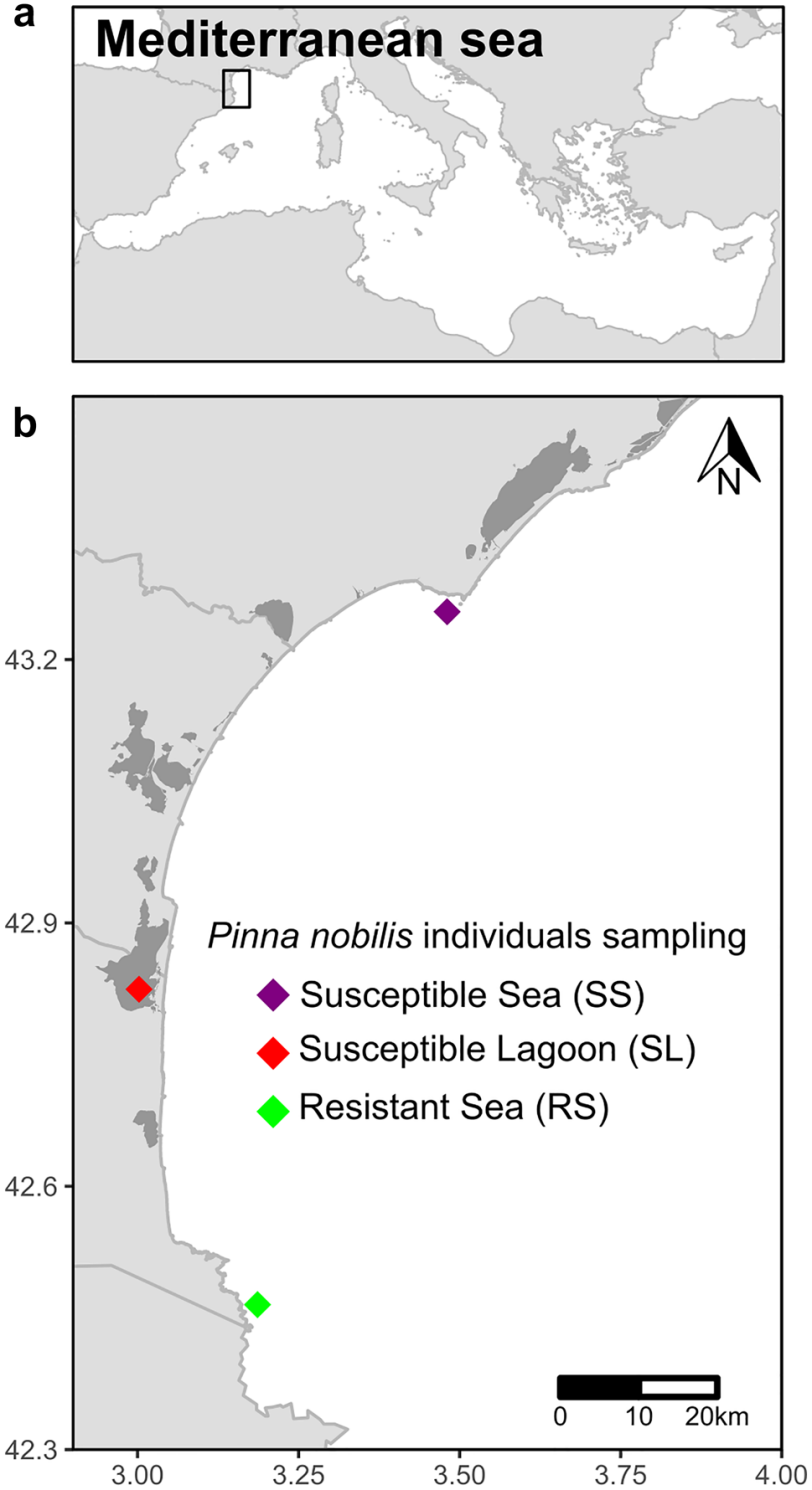
Sampling location of *P. nobilis* individuals resistant or susceptible to *H. pinnae* parasite. (**a**) *P. nobilis* species is endemic to the Mediterranean Sea. (**b**) Individuals were sampled in three different locations in the Mediterranean Sea: Resistant from the sea were sampled in Peyrefite Bay (green), Susceptible from the Sea were sampled in Agde (Purple) whereas Susceptible from the Lagoon were sampled in Leucate (Red). RS, Resistant from Sea; SS, Susceptible from Sea; SL, Susceptible from Lagoon.

In the bay of Peyrefite, the population of *P. nobilis* was estimated at around 630 individuals (Fig. 1a,b), before the beginning of the mass mortality event that occurred in 2018^21^. After summer 2018, only four live individuals remained. Considering that individuals were close to each other during the infection by *H. pinnae*, we hypothesized that they were likely exposed to the parasite. Interestingly, these individuals did not present any symptoms of infection (e.g. lack of response to stimuli and slow closing of the valves).

Since *P. nobilis* individuals can be confused with other species that are not affected by the parasite (i.e., *P. nobilis-Pinna rudis* hybrids and *P. rudis*), we started by classifying the species of the survivors. According to the results based on the COI mtDNA and microsatellites screening, all sampled individuals in this study belonged to *P. nobilis* species. Next, to determine whether these individuals were resistant (i.e. possessing mechanisms to reduce the establishment and/or to clear the pathogen following establishment) or tolerant (i.e. possessing mechanisms which limit somatic damage without decreasing pathogen load) to *H. pinnae*, we investigated the presence of the parasite. A PCR amplification of a species-specific fragment of the small subunit ribosomal DNA (SSU rDNA)^14^ with two positive controls did not detect the presence of *H. pinnae*^14^. This, together with the absence of the infection’s symptoms, demonstrates that individuals were most likely resistant instead of tolerant. Moreover, we checked the presence of 18S rDNA (SSU rDNA) gene transcripts (LC338065) specific to *H. pinnae* in the de novo transcriptome of our samples to confirm that resistant fan mussels had been in contact with the parasite. Interestingly, we found specific transcripts of 18S rDNA in two of our three resistant individuals as well as in two susceptible individuals sampled along the coast in the region of Agde. For the latter two, the presence of the 18S rDNA transcripts suggests an initial state of contamination by the parasite. In fact, all susceptible individuals from Agde used in this study died a few months later due to the infection by *H. pinnae*. For resistant individuals, the presence of the 18S rDNA suggests that resistant individuals co-exist with the parasites and further proves their resistance.

### De novo transcriptome assembly of *P. nobilis*, annotation, and quality check

To better understand the physiology of this resistance, we performed a transcriptomic analysis in which we compared *P. nobilis* putative resistant individuals with susceptible individuals. Resistant individuals were sampled along the sea coast in the Bay of Peyrefite (marked here as «RS»). We sampled eight susceptible individuals: four found along the sea coast close to Agde (marked «SS») and four susceptible individuals sampled in Leucate lagoon (marked «SL») (Fig. 1a,b and Table S1). Populations of *P. nobilis* in the area of Leucate lagoon and along the sea coast close to Agde did not show signs of infection by *H. pinnae* at the time of sampling. Because no *P. nobilis* reference transcriptome was available, we began by constructing one as a baseline to qualify and calibrate differences in the expression. Each individual was sequenced and the reads from all the samples were combined to form a unified meta-sample. Finally, a de novo transcriptome was assembled. After quality control of the pipeline, the final transcriptome assembly consisted of 269,285 unique transcripts (for a total of 451,934,494 bases) with lengths ranging from 132 to 59,616 bases with an average of 1678 bases (521 transcripts under 200 bases, 118,667 over 1000 bases, and 2278 over 10,000 bases). An estimated 80,790 transcripts had an open reading frame (covering, on average, 35.6% of the transcript). A single-copy ortholog (BUSCO) assessment of transcriptome completeness identified 5084 (96%) complete BUSCOs^24,25^. These parameters confirm that the *P. nobilis* transcriptome is well assembled and complete. Among the 269,285 transcripts included in the assembly, 125,180 (46%) received an annotation.

### The three putative-resistant *P. nobilis* individuals have a specific gene expression profile

Using this de novo transcriptome, we investigated levels of gene expression across all individuals (three RS, four SS, four SL) (Table S2). We found 1925 differentially expressed genes (DEGs) (1160 up-regulated and 765 down-regulated) between RS and SS; and 1297 DEGs (889 up-regulated and 408 down-regulated) between RS and SL. Fewer DEGs (i.e., 245) were found between SS and SL (18 up-regulated and 227 down-regulated).

**Figure 2:**
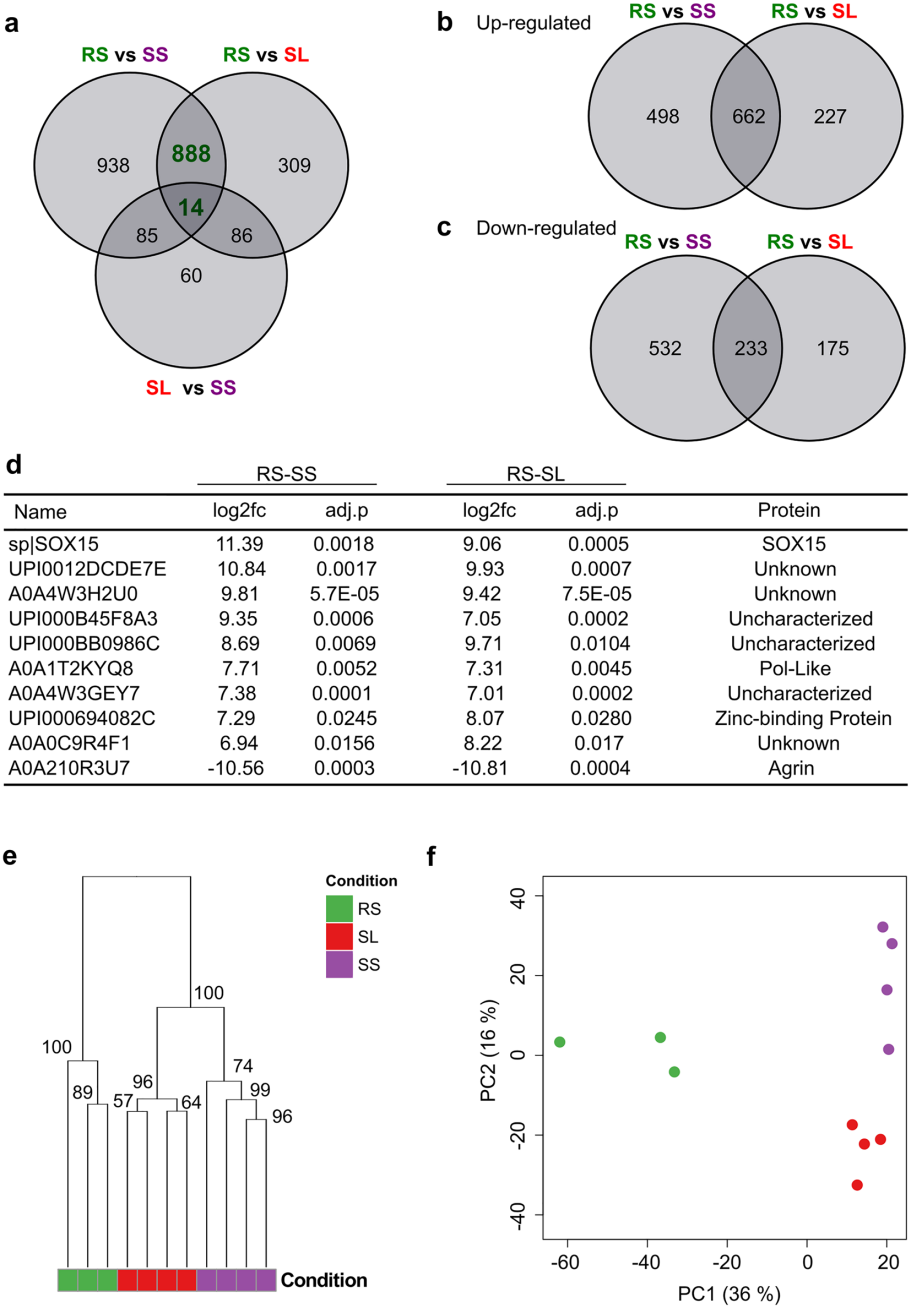
Resistant *P. nobilis* shows a specific differential expression genes profile. (**a**) Venn diagram showing the overlap of DEGs identified in RS vs SS, RS vs SL and SL vs SS. (**b**) Venn diagram showing the overlap of DEGs overexpressed in the two comparisons RS vs SS and RS vs SL. (**c**) Venn diagram showing the overlap of DEGs down-regulated in the two comparisons RS vs SS and RS vs SL. (**d**) 10 genes mostly significantly differentially regulated in both comparisons RS vs SL and RS vs SS and the protein associated. (**e**) Hierarchical clustering of samples (Ward’s method). The 1000 genes with highest variance were kept as dimensions. The percentage above each cluster divide represents the stability of the group as estimated by multiscale bootstrap resampling. Please note that the percentage that divides the clusters resistant with susceptible is equal to 100%. (**f**) Principal component analysis of samples. The 1000 genes with highest variance were kept as dimensions. RS, Resistant from sea; SS, Susceptible from sea; SL, Susceptible from lagoon.

The set of DEGs in common between the three comparisons was then investigated (Table S2). 902 DEGs were in common between RS vs SS and RS vs SL (Fig. 2a) for which 895 were positively correlated and 7 were negatively correlated. Among the 895 positively correlated genes, 662 were over-expressed in resistant vs susceptible individuals and 233 were down-regulated (Fig. 2b,c). In both comparisons (RS vs SL and RS vs SS), the 10 genes that were the most significantly differentially regulated (with the highest absolute value of log_2_FC) represented genes coding for Sox15, Agrin, a Pol-like protein, Zinc-binding protein, and 6 unknown genes (Fig. 2d). Together, this suggests the existence of a specific gene expression signature in *P. nobilis* that is resistant to *H. pinnae* in comparison to susceptible individuals.

We also found 100 DEGs in common between RS vs SL and SS vs SL (Fig. 2a). Among those, 90 are commonly overexpressed and 4 are commonly down-regulated (Fig. S1a and b). We notably found genes coding for a heat-shock protein, transcription regulators proteins, and a protein involved in a nutrient reservoir (Fig. S1c).

To verify whether this phenotype signature effectively represents the resistant status of the individuals as opposed to differences in sampling locations, we performed a hierarchical clustering and a PCA analysis. Both analyses showed three distinct clusters: the three resistant RS (in green), the four SS (in purple) and the four SL (in red) (Fig. 2e,f). Interestingly, the PCA revealed a clear separation between resistant (RS) and susceptible individuals (SS and SL) along the PCA1 axis which encompassed 36% of the variance (Fig. 2f). The coastal and lagoonal individuals were also separated along the PCA2 axis (16% of the variance), suggesting a difference in expression due to environmental conditions (Fig. 2f). Lastly, the three individuals RS showed a very similar gene expression pattern between them. Overall, both hierarchical and non-hierarchical clustering algorithms demonstrated that the most significant difference between individuals relied on the *H. pinnae* resistant phenotype. Some ecological differences (namely coastal vs. lagoonal) were also visible but are less important since they carried less variance in the overall analysis.

### The immune system and cell architecture are related to *P. nobilis* resistance to *H. pinnae* infection

**Figure 3:**
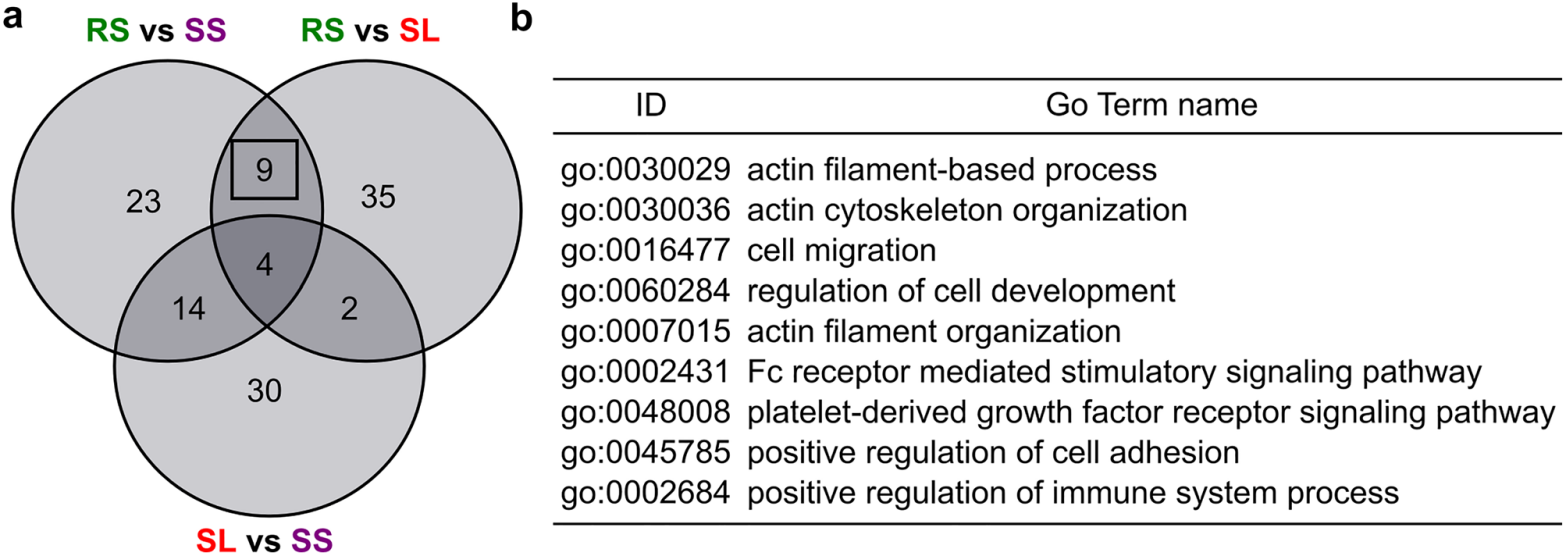
Gene ontology set analysis. (**a**) Venn diagram showing the overlap of GO terms that were enriched in DEGs identified in RS vs SS, RS vs SL and SL vs SS. (**b**) Common GO terms only between RS vs SS and RS vs SL. RS, Resistant from sea; SS, Susceptible from sea; SL, Susceptible from lagoon.

To determine the biological processes that correspond to the DEGs in our three previous comparisons (RS vs SS, RS vs SL, and SL vs SS), we performed a Gene Ontology (GO) enrichment analysis (Table S3). As shown in a Venn diagram analysis, 9 biological processes are exclusive to the comparisons RS vs SS and RS vs SL (Fig. 3a,b). These biological processes correspond mainly to cell architecture (e.g., Actin cytoskeleton organization, positive regulation of cell adhesion) and immunity (FC receptor mediated stimulatory signaling pathway, platelet-derived growth factor receptor signaling pathway, and positive regulation of immune system processes). A closer inspection of the DEGs found within these two main biological processes (Table S4) underlined a set of genes that were highly over-expressed both in RS vs SS and RS vs SL (log_2_FC > 4). Concerning the immunity process, *PTPRJ* and *PTPRC* code for two protein tyrosine phosphatase receptors, and *IRG1* is the immune responsive gene 1. We also found *TLR4*, *LEG9*, *TRAF2*, *FYN* that coded for proteins known to be involved in bivalve’s pathogen recognition (such as TLR4 and LEG9) and in signal transduction (such as TRAF2)^26,27^. Moreover, a closer look at the biological process “positive regulation of cell adhesion” revealed three genes coding for integrins (*ITA5*, *ITB1,* and *ITB3*) known to be expressed in hemocytes^27^.

Two biological processes are exclusive to the comparisons RS vs SL and SL vs SS: go:0043405 (regulation of MAP kinase activity) and go:0034097 (response to cytokine).

To conclude, this analysis suggested that the immune system and cell architecture are linked to resistance to *H. pinnae* infection.

## Discussion

This study identified four *P. nobilis* individuals that survived exposure to the *H. pinnae* parasite in a bay that initially hosted more than 630 individuals (about 0.6% survivorship). Our results assume that those individuals were in contact with the parasite and that they were resistant to it rather than tolerant. We conducted the first comparative transcriptomic study between *P. nobilis* individuals that were susceptible and resistant to the parasite, in order to understand the potential etiology of the disease and to determine how some individuals escape pathogenicity associated with *H. pinnae* infection. As no transcriptome was available for this species, we performed a de novo transcriptome assembly and annotated the expressed genes. A RNA-Seq comparative study between susceptible and resistant individuals revealed specific genes differentially expressed between those two groups. Importantly, those DEGs correspond to both cell architecture and immunity biological processes.

To understand the mechanisms behind the survival of *P. nobilis* to *H. pinnae* infection, we checked if the parasite was present in the mantle tissue of survivors from the Bay of Peyrefite since this tissue is commonly used to detect the parasite^14^. PCR amplification was unable to detect the parasite in any of these individuals, demonstrating a resistance to the parasite. As the transcriptomic approach is more sensitive, we verified whether we could find parasite-specific transcripts of 18S rDNA in the global transcriptome screening to verify the assumption that the resistant individuals had been in contact with the parasite. Interestingly we confirmed the presence of the parasite in two of the three resistant individuals. We were unable to detect the parasite transcript in the third resistant individual. However, as all three were found alive in a population of approximately 630 individuals, all of which had been infected and killed by the parasite^21^, and because they were in close proximity to dead individuals and were not found in a different habitat (personal communication), it is highly unlikely that the third surviving individual did not come in contact with the parasite. Surprisingly, we also found the 18S rDNA parasite-specific transcripts in the global transcriptome of two susceptible individuals sampled along the sea coast of Agde. This may be explained by the fact that these individuals died soon after (late 2019) the sampling (September 2019) due to exposure to *H. pinnae*.

Transcriptome sequencing offers a starting point and a cost-effective method for characterizing the gene set in a non-model species and in our case, allows for the exploration of the underlying mechanisms involved in *P. nobilis* resistance to *H. pinnae*. A BUSCO analysis, with a score of 96%, revealed excellent completeness and integrity of our transcriptome (i.e., fragmentation of genes) in comparison with molluscan libraries^28^. Transcriptomes from different species of mollusks have shown that the expected number of transcripts ranges from approx. 34,794 to 394,251^29^. In our study, we found 269,285 transcripts in total, a number within the higher range of what is typically found for mollusks. This demonstrates the high quality of our transcriptome. However, only 46% of transcripts were annotated. This high proportion of non-annotated transcripts is not unusual in transcriptome projects for invertebrate taxa (^30,31^, including mollusks^29,32–34^). The lack of detectable sequence orthologues in the public databases may be due to several factors, including taxonomically restricted genes (e.g., orphan genes), novel isoform transcripts or protein-coding genes, non-functional coding sequence regions, and poor quality of the sequences themselves or the assembly procedures performed^35,36^. For mollusks, various studies have described the emergence of numerous genes and gene families, which are either present in different molluscan lineages or are restricted to one^29,37,38^. Overall, we expected the weak annotation level in *P. nobilis*. However, this does not impact the scope of research in our study.

In this RNA-seq study, we compared three populations: a group of resistant individuals found along the sea coast in the bay of Peyrefite, another of four susceptible individuals found along the sea coast in Agde and a group of four other susceptible individuals collected from the lagoon of Leucate. Unfortunately, we could not increase the number of resistant individuals in our study as the number of survivors after infection was very low, and no other live individuals were reported at the time of this study. The three individuals involved in this study, along with the one we failed to sample without too much invasiveness, were the only ones found alive in the bay and in surrounding areas at the time of sampling. Moreover, in this study, we were unable to compare susceptible and resistant individuals found in the same area as we did not sample individuals in the bay of Peyrefite before the pandemic and thus did not have access to the transcriptome of susceptible individuals in this bay. It’s clear that targeted monitoring and sampling efforts in a remaining healthy population of *P. nobilis* would lead to a more accurate study. However, despite the small number of individuals screened in each case and the fact that resistant and susceptible individuals were not found in the same location, a hierarchical clustering of the expression profile of each individual demonstrated a higher divergence of all resistant individuals compared to susceptible ones. This indicates a major effect of resistant vs susceptible phenotypes on DEGs in comparison with the effect of sampling location which shows reduced divergence when comparing two susceptible populations.

Mollusks do not have any adaptive immunity and rely solely on innate or non-specific immunity for host defense^26^. The bivalve defense system includes several layers of physical and biological barriers. The physical barrier for infection is provided by the skin and its mucosal layer, which entraps microbes and facilitates their elimination through ciliary activity^39,40^. Internal defense is governed by innate immunity: hemocytes and dissolved humoral factors of the plasma work in a complementary fashion to neutralize invading organisms^39,40^.

To determine the underlying mechanisms involved in *P. nobilis* resistance to *H. pinnae*, we investigated which biological processes are enriched in resistant individuals compared to susceptible ones. We found biological processes involved in the immune response including: an FC receptor mediated stimulatory signaling pathway, a platelet-derived growth factor receptor signaling pathway and positive regulation of immune system processes. Interestingly, two innate immune receptors *TLR4* and *LEG9* are overexpressed in resistant bivalves coding for Toll-like receptors and Galectin proteins respectively. Toll-like receptors have been identified and implicated in all mollusks studied so far, including *Chlamys farreri*^41^, *Crassostrea virginica*^42^, *Ruditapes philippinarum*^43^, *Mytilus galloprovincialis*^44^ and *Crassostrea gigas*^45,46^. Galectin is a family of lectins which are known to be involved in the immune systems of marine mollusks and *LEG9* galectin is expressed in *Pecten maximus* hemocytes^27^. Since mollusks do not have an adaptative immunity, they instead possess many innate immune receptors that may be involved in the recognition of parasites^26^. Thus, TLR4 and LEG9 proteins may provide great specificity in immune recognition of *H. pinnae* and thus its elimination, which may explain the resistance to *H. pinnae* observed in the three *P. nobilis* individuals (RS) that had been exposed to the parasite. Among the nine common biological processes enriched in RS vs SS and RS vs SL, and absent in SL vs SS, we also found six biological processes involved in cell architecture. Because it has been suggested that the infectious process stops if a pathogen is successfully neutralized by the host at a portal of entry^47–49^, such as the physical barrier provided by the skin and the mucosal layer, our results suggest that cell architecture, and potentially that of the epithelium, could be involved in the capacity of *P. nobilis* to resist *H. pinnae* infection. However, while Box et al.^20^ showed that infection of the fan mussel by *H. pinnae* prevented the antioxidant system from being activated and increased oxidative damage, we could not find a differential expression of genes coding for antioxidant enzymes in our analysis^27^. Our data suggest that the antioxidant system may not play a role in the resistance.

To conclude, this study proved the existence of *P. nobilis* that are resistant to *H. pinnae*. This finding provides hope that new solutions will be found to prevent the extinction of this species such as applying in vitro reproduction between resistant individuals to grow resistant spat and thus repopulate devastated fan mussel populations^16^. However, efforts have to be made to improve their complete in vitro reproduction (i.e. in aquarium)^16,50^.

Moreover, this study provides a valuable RNA-seq resource of *P. nobilis* as well as a first insight into understanding their molecular process of resistance against *H. pinnae*. This study represents a first and crucial step to understanding how global changes may affect the spreading of infectious novel pathogens (here *H. pinnae*) in naïve host species (here *P. nobilis*).

## Methods

### Sampling collection

In this study, we worked on *P. nobilis* individuals in three locations: individuals sampled in Peyrefite bay, individuals sampled along the sea coast in the region of Agde and individuals sampled in the lagoon of Leucate.

Since the beginning of the pandemic, fan mussel populations have been monitored annually to record the spread of the disease, mortality rates within each population, and the presence of survivor individuals. In the Gulf of Lion, the first signs of infection by *H. pinnae* were reported in July 2018 in the bay of Peyrefite and then spread rapidly throughout the entire coast the next year and moved even further the following year. Before the pandemic, this population was estimated to be about 630 individuals (Peyran et al., *in revision*). At the end of summer 2018, only four individuals remained healthy, and were still alive in summer 2019. In 2019, individuals along the sea coast of Agde and in the lagoon of Leucate were sampled, before the spread of the parasite in these areas. By late 2019, the Agde population was decimated by *H. pinnae* and, as sampled individuals died, they were considered as susceptible to the disease. Noticeably, not a single individual was observed in the Agde population after the population was hit by *H. pinnae*. Throughout all of the Mediterranean Sea, fan mussel populations found in lagoons appear to be less affected by the parasite, which is perhaps linked to the environmental conditions in these habitats that do not favor the proliferation of *H. pinnae*^16^. Populations in Leucate lagoon are no exception and, even while some mortality caused by *H. pinnae* was reported in the channels connecting the lagoon to the open sea, there are still dense and healthy populations inside the lagoon. Sampled individuals in the lagoon of Leucate are thus still alive but, as resistant individuals are very scarce, it is more than likely that they are all susceptible to the parasite.

To resume, we sampled mantle tissue (biopsy of ~ 1 cm^3^) in situ (on SCUBA) from three individuals which were considered as resistant, found along the coast of France’s Peyrefite Bay (referred to below as «RS» for Resistant from the Sea), from four individuals considered as susceptible because they are found in a non-affected area which were found on the coast of France’s Cap d’Agde and (referred to below as «SS» for Susceptible from the Sea), and four others found in a lagoon not infected by *H. pinnae* (Leucate Lagoon, France) also considered as susceptible (referred to below as «SL» individuals for Susceptible from the Lagoon) (Fig. 1a,b and Table S1). Sampling of mantle tissue in non-lethal. Biopsies were put directly in RNAlater (RNAlater R0901; Sigma-Aldrich) and stored at − 20 °C. From a subsample of these biopsies, we first performed DNA extraction to confirm that individuals belonged to the *P. nobilis* species and to check for the presence of the parasite. We also performed RNA extraction from another subsample for transcriptomic analysis.

### DNA extraction, species confirmation, and detection of *H. pinnae*

A recent study demonstrated that hybridization among *P. nobilis* and its sister species *Pinna rudis* exists and that these hybrids can survive *H. pinnae* infection^51^. To confirm the species of individuals, we used cytochrome c oxidase subunit I (COI) mtDNA and microsatellite markers. Mitochondrial DNA such as COI sequences are usually used for species identification but, because it is maternally inherited, it is not informative enough to detect hybrids. Thus, microsatellite markers were also used as they were shown to present good cross-species transferability (Peyran et al.^54^) but with different allele sizes which allow for discrimination between the two species. Due to limited or lack of tissue sample for certain individuals, we could only do these experiments for seven individuals (two RS individuals, one SS individual, and four SL individuals).

#### DNA extraction

Extractions of genomic DNA were performed using the Gentra Puregene Tissue Kit (Qiagen, Hilden, Germany) following manufacturer’s instructions. Additional 15 *P. nobilis* individuals (Peyrefite bay, Banyuls, France) and 23 *P. rudis* (Cabrera, Balearic Island, Spain) were added to the study for genotype comparison and DNA was extracted using the QIACube robot (Qiagen, Hilden, Germany) according to manufacturer’s instructions.

#### Mitochondrial sequences

A species-specific set of primers developed by Katsares et al.^52^⁠ were used for amplification of a fragment of the COI mtDNA gene. PCRs were performed using Taq PCR Core Kit (Qiagen, Hilden, Germany) in a volume of 23 µL containing 2.5 µL of 10× PCR Buffer, 2 µL of MgCl_2_ (25 mM), 2.5 µL of dNTP mix (2 mM), 1 µL of each primer (10 µM), 0.1 µL of TAQ DNA polymerase (5 U/µL), 11.9 µL of RNA free water and 2 µL of DNA template (5 ng/µL). Thermal cycling of PCR consisted of an initial denaturing step of 3 min at 94 °C, followed by 40 cycles of amplification (1 min at 94 °C, 1 min at 54 °C, and 1 min at 72 °C) and a final extension step of 3 min at 72 °C. PCR products were then sent to GenoScreen (Lille, France) to be sequenced on an Applied Biosystems 3730 Sequencer.

#### Microsatellite loci

All samples were genotyped using 10 microsatellite markers that were shown to present clear amplification for both species (3.2, 3.5, 4.3, 4482, 5017, 6980, 11,847, 14,331, 15,415, 15,584)^53,54^⁠. PCRs were performed using a Type-it Microsatellite PCR kit (Qiagen, Hilden, Germany) and scored following the procedure described in^54^⁠.

#### Data analysis: species confirmation

The species of each sample was verified by comparison of the obtained COI sequences (700 bp) with the reference sequences in the GenBank database using the BLAST-n algorithm (https://blast.ncbi.nlm.nih.gov/Blast.cgi). Individuals were assigned to a species level when the identity of the compared sequences was at least 98%. A Principal Coordinate Analysis (PCoA) was performed, using GenAlex^55^, to compare RS samples to individuals from *P. nobilis* and *P. rudis*, based on their genotype at the 10 microsatellite loci.

#### Detection of *H. pinnae*

The presence of *H. pinnae* was investigated through the amplification of a specific fragment of 600 bp of the small subunit ribosomal DNA (SSU rDNA), using primer pairs: HPN-F3/HPN-R3, developed by^14^). PCRs were performed using the same protocol as previously described for amplification of the fragment of the COI mtDNA gene and using the following thermal cycling program: an initial denaturing step of 10 min at 94 °C, followed by 40 cycles of amplification (1 min at 94 °C, 1 min at 55 and 1 min at 72 °C) and a final elongation step of 10 min at 72 °C. PCR products were separated on a 2% agarose in a 0.5× TBE buffer gel, stained with 1% of ethidium bromide, and including 100 bp DNA ladder size standard. Visualization was performed under UV light. Two infected individuals from Peyrefite that were sampled during the mass mortality event in 2018 and which already demonstrated the presence of *H. pinnae,* were used as a positive control for this analysis.

### RNA extraction, RNA‐Seq library preparation, and sequencing

Lysis of tissue was done using MP Biomedicals™ Instrument FastPrep-24™ 5G (fisher scientific- reference: 15,260,488). The RNA was automatically extracted using Maxwell^®^ RSC Instrument (Promega, reference: AS4500) and Maxwell^®^ RSC simplyRNA Tissue Kit (Promega-reference: AS1340). RNA‐Seq libraries were generated with TruSeq Stranded mRNA Sample Preparation Kit (Illumina) from 400 ng of total RNA according to manufacturer's instructions (subcontracted GenomEast). Surplus PCR primers were removed using AMPure XP beads (Beckman Coulter). Final cDNA libraries were checked for quality and quantified using capillary electrophoresis. Libraries were loaded in the flow cell at 2 nM. Clusters were generated in the Cbot and sequenced on an Illumina HiSeq 4000 as paired‐end 2 × 100 base reads (stranded protocol). Transcriptomic and statistical analyses are described in Supplementary Methods.

### Compliance with ethical standards

The sampling was non-lethal and approved by the DREAL (Direction Régionale de l’Environnement, de l’Aménagement et du Logement) of Occitanie (prefectural order n°2018-s-24).

## Supplementary Information

Supplementary Information 1.

Supplementary Table S2.

Supplementary Table S3.

Supplementary Table S4.

## Data Availability

RNA-Seq FASTQ raw sequencing data files as well as *Pinna nobilis*’ de novo transcriptome are available at Dryad under 10.5061/dryad.xwdbrv1dm.
