# Supplementary material for: RNA-Seq comparative study reveals molecular effectors linked to the resistance of Pinna nobilis to Haplosporidium pinnae parasite
Source: Sci Rep. 2022 Dec 8;12:21229. doi: 10.1038/s41598-022-25555-x (PMC9731998; doi:10.1038/s41598-022-25555-x)
Supplement: Supplementary file 1 — Supplementary Information 1. [file 41598_2022_25555_MOESM1_ESM.docx]

**Supplementary information**

**Additional figures**


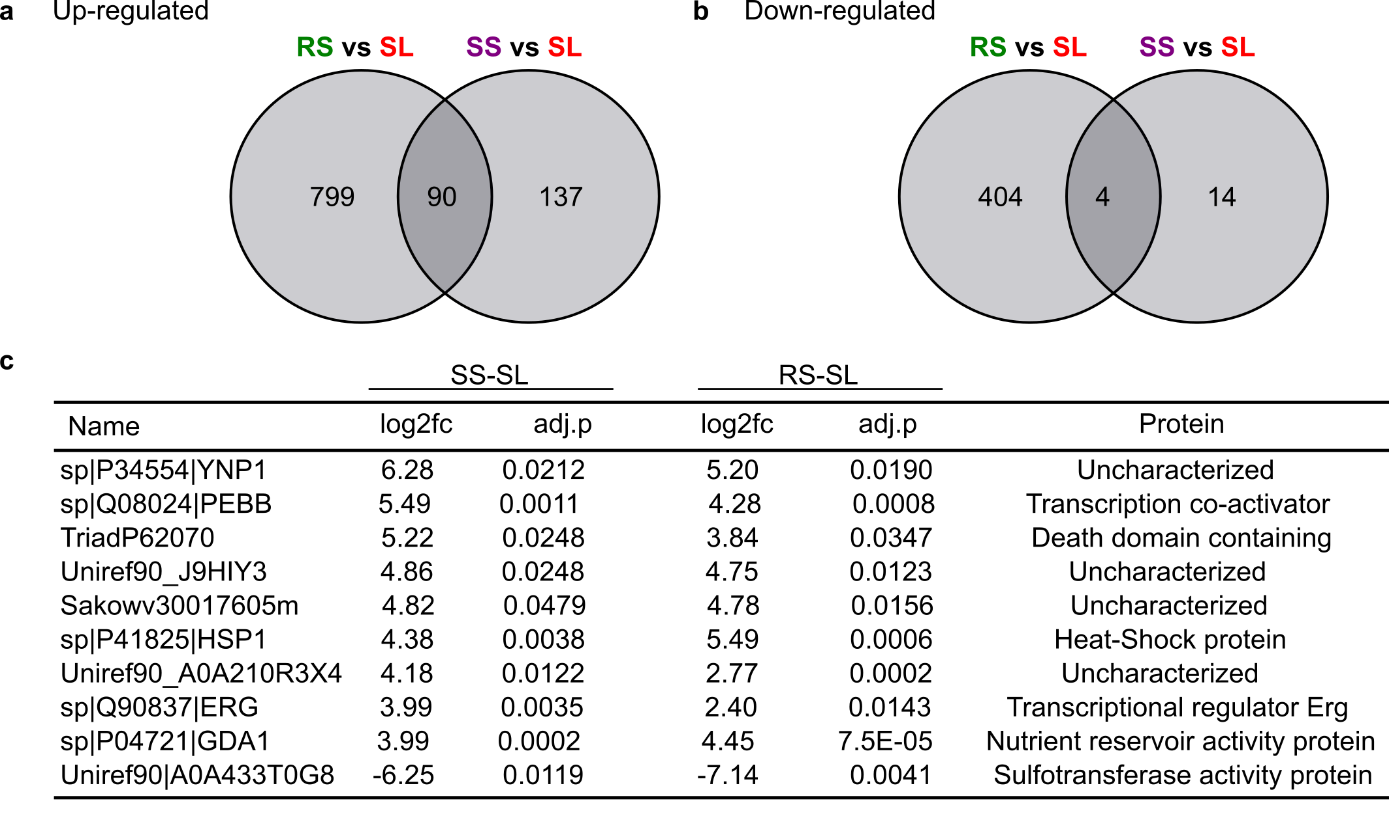


Figure S1- Individuals of *P. nobilis* sampled in the lagoon show a specific DEGs profile

(a) Venn diagram showing the overlap of DEGs up-regulated in the two comparisons RS vs SL and SS vs SL.

(b) Venn diagram showing the overlap of DEGs down-regulated in the two comparisons RS vs SL and SS vs SL.

(c) 10 genes mostly significantly differentially regulated in both comparisons RS vs SL and SS vs SL and the protein associated.


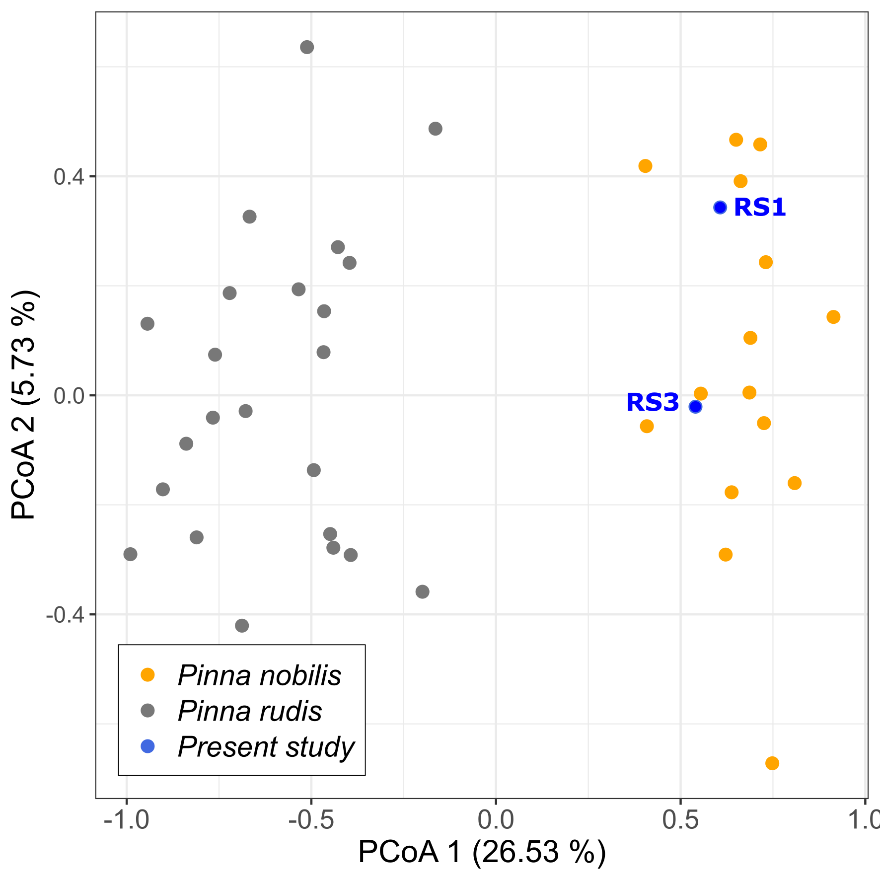


Figure S2. RS individuals are identified as *P. nobilis*.

Principal Coordinate Analysis (PCoA) confirms that RS (blue dots) individuals clusters along the axis PCoA1 together with *P. nobilis* individuals (orange dots) rather than with *P. rudis* individuals (grey dots). The species of each sample was confirmed using cytochrome c oxidase subunit I (COI) mtDNA and microsatellite markers.

**Additional tables**

Table S1. Basic information of the 11 *Pinna nobilis* individuals used for transcriptomic. From those 11 individuals, RS-1, RS-3, SL-1, SL-2, SL-3, SL-4 and SS-3 were used for DNA extraction species confirmation, and detection of *H. pinnae*.

| **Sampling date** | **Sample Name** | **Location** | **Maximum width of the shell (cm)** | **Minimum width of the shell (cm)** | **Unburied length (cm)** | **Total**  **(cm)** | **Depth**  **(m)** | **Habitat** | **Status of P. nobilis susceptibility to *Haplosporidium pinnae***  **(Resistant or Susceptible)** |
| --- | --- | --- | --- | --- | --- | --- | --- | --- | --- |
| 30/07/2019 | RS-1 | Peyrefite | 20 | 12 | 27 | 59,5 | 5,7 | *Posidonia oceanica* meadow (Neptune grass) | Resistant |
| 30/07/2019 | RS-2 | Peyrefite | 18 | 12 | 21 | 48 | 6,1 | *Posidonia oceanica* meadow (Neptune grass) | Resistant |
| 30/07/2019 | RS-3 | Peyrefite | 16 | 12 | 22 | 48,5 | 7,2 | *Posidonia oceanica* meadow (Neptune grass) | Resistant |
| 09/09/2019 | SL-1 | Salses-Leucate (south) | 18 | 12 | 24 | 53,2 | 1,5 | Shell mud + *Zostera noltii* (Seagrass) | Susceptible |
| 09/09/2019 | SL-2 | Salses-Leucate (south) | 17 | 13 | 24 | 52,5 | 1,5 | Shell mud + *Zostera noltii* (Seagrass) | Susceptible |
| 09/09/2019 | SL-3 | Salses-Leucate (south) | 17 | 12 | 23 | 50,8 | 1,6 | Shell mud + *Zostera noltii* (Seagrass) | Susceptible |
| 09/09/2019 | SL-4 | Salses-Leucate (south) | 16 | 12 | 24 | 51,8 | 1,8 | Shell mud + *Zostera noltii* (Seagrass) | Susceptible |
| 17/09/2019 | SS-1 | Agde | 16 | 14 | 16 | 38 | 4,1 | Matte + *Caulerpa racemosa* (sea grapes) | Susceptible |
| 17/09/2019 | SS-2 | Agde | 13 | 10 | 18 | 39,8 | 4,2 | Matte + *Caulerpa racemosa* (sea grapes) | Susceptible |
| 17/09/2019 | SS-3 | Agde | 17 | 17 | 13 | 32,9 | 4,2 | Matte + *Caulerpa racemosa* (sea grapes) | Susceptible |
| 17/09/2019 | SS-4 | Agde | 8 | 8 | 14 | 29,6 | 3,8 | Matte + *Caulerpa racemosa* (sea grapes) | Susceptible |

Table S2- List of genes identified as differentially expressed for each named statistical contrast (RS vs SS, SL vs SS and RS vs SL) (ordered by decreasing absolute value of log2 fold change).

Table S3- Top 50 GO biological process categories of differentially expressed genes for each comparison (RS vs SS, SL vs SS and RS vs SL).

Table S4- Differentially expressed genes involved in the top 50 GO biological processes for each comparison (RS vs SS, SL vs SS and RS vs SL).

Table S5- Categories representing BUSCO quantitative assessment of the completeness in terms of expected gene content of the assembly: Complete (C) and single-copy (S), Complete and duplicated (D), Fragmented (F), or Missing (M)

| **N** | **Type** |
| --- | --- |
| 5,084 | Completed BUSCOs (C) |
| 1,431 | Complete and single-copy BUSCOs (S) |
| 3653 | Complete and duplicated BUSCOs (D) |
| 21 | Fragmented BUSCOs (F) |
| 190 | Missing BUSCOs (M) |
| 5,295 | Total BUSCO groups searched |

Table S6- Number of transcripts annotated (at least once) by each process

| **Process** | **Number of transcripts** |
| --- | --- |
| HMMER | 74,769 |
| Infernal | 1,160 |
| LAST | 123,459 |

**Additional Methods**

**Generation of a *de novo* transcriptome and annotation**

- Read Quality

The quality of raw reads was assessed using the FastQC quality control tool (http://www.bioinformatics.babraham.ac.uk/projects/fastqc), version 0.11.8.

- Estimation of Contamination

Sample contamination was assessed using the fastq_screen quality control tool (http://www.bioinformatics.babraham.ac.uk/projects/fastq_screen; version 0.13.0) on the FASTQ file of the first mate of each sample. For *P. nobilis*, the bowtie2 index was generated using the *P. nobilis* genome ^1^. No significant contamination was observed in this dataset. The same tool was used to check for the presence of specific *Haplosporidium* species on the 18S rDNA (SSU rDNA) gene sequence (LC338065) in the samples ^2^.

- Generation of a Transcriptome

All the first (respectively second) mate reads from the 11 samples were combined into a unified “R1” (respectively “R2”) FASTQ file to generate a “meta-sample”. A first transcriptome was then generated from these two FASTQ files using the multi-kmer multi-assembler approach of the Oyster River Protocol ^3^ (commit version 85f7ca002d13aa26ebe4a4906a1e58befe54bacd), with parameters TPM_FILT=0.1, STRAND=RF, and LINEAGE=mollusca_odb10.

The Oyster River Protocol assembly consists of 306,287 transcripts (for a total of 482,008,288 bases) with lengths ranging from 131 to 59,616 bases (average: 1,573 bases, 545 transcripts under 200 bases, 123,866 over 1,000 bases, and 2,484 over 10,000 bases). 86,003 are estimated to have an open reading frame (covering, on average, 36.6 % of the transcript). The quality of the *de novo* assembly was assessed using The TransRate package (^4^ version 1.0.3)). The quality score is assessed using read and contig data and estimates individual contig and overall assembly quality. The score of this first transcriptome was 0.244 (better than 50 % of published de novo assembled transcriptomes) ^4^.

We then created a final transcriptome by considering only the “good transcripts” as those which pass the TransRate cutoff 0.0695 (^4^ version 1.0.3). The score of assembly of this final transcriptome according to TransRate becomes 0.355.

The completeness of the final transcriptome was estimated using BUSCO ^4,5^ (version 4.0.5) with options -m transcriptome and -l mollusca_odb10 (dataset date: 2019/11/20). The results are simplified into categories of Complete (C) and single-copy (S), Complete and duplicated (D), Fragmented (F), or Missing (M) BUSCOs. According to BUSCO, completeness was C:96.0 % [S:27.0%, D:69.0%], F:0.4%, M:3.6%, n:5,295 (values for each type reported in Table S5).

- Annotation

The resulting transcriptome was annotated using the dammit *de novo* transcriptome annotator (http://dib-lab.github.io/dammit/ version 1.2) with parameters --busco-group metazoa and –full. Dammit annotates de novo transcriptomes by blasting the sequences into available databases. The output of blast results is the scoring of an alignment by an "Expect value" (e-value). Here, an e-value cut-off of 10^−5^ was selected to filter the annotations. Among the 269,285 transcripts of the assembly, 125,180 received an annotation with an e-value below 10^−5^ (disregarding the gene model information generated by TransDecoder), for a total of 503,317 annotations. Table S6 shows the number of transcripts annotated (at least once) by each process.

The databases used with dammit were: Pfam-A (version 28.0), Rfam (version 12.1), Swiss-Prot (version 2020_02), UniRef90 (version 2020_02), and OrthoDB (version 8).

**Genes Differentially Expressed in *P. nobilis* resistant to *H. pinnae.***

- Quantification and summarization

Transcript expression quantification was performed from the raw read data (FASTQ files) using Salmon with parameters --gcBias --seqBias --mimicBT2 and the *de novo* transcriptome generated during this project. Mapping proportions range from 86.8 % to 92.8 % with a median of 90.8 %.

They were then summarized as gene counts using function tximport from package tximport ^6^ (with parameters type="salmon" (software) and countsFromAbundance="lengthScaledTPM"). Salmon is a software to map reads on the transcriptome. The mapping from transcript to gene was based on the dammit-generated annotations: annotations with an e-value above 10^-5^ were discarded. Among the remaining annotations for a given transcript, those resulting from Infernal (if none, from LAST, and, if none, from HMMER) were selected. If matches to different databases were present, then the ones from Swiss-Prot were selected if present, otherwise, UniRef90, and finally OrthoDB. In the case of multiple matches to the same database, the match with the best score was selected. The resulting unique annotation was set as the gene name for this transcript. Additionally, the species-identifying part of Swiss-Prot identifiers was dropped to merge corresponding transcripts from different species.

- Filtering

Genes that did not have more than 0.281 counts per million counts in at least 3 samples were filtered out. 14,596 genes out of 38,379 (38 %) were considered not expressed and were excluded from further analyses. The total count number (after filtering) for each sample ranges from 35.5 to 65.1 million.

- Normalization

To generate the normalized signals, the effective library sizes were first computed using estimateSizeFactors from package DESeq2 ^7^ (https://bioconductor.org/packages/release/bioc/vignettes/DESeq2/inst/doc/DESeq2.html).

The raw count values were then transformed using a variance stabilizing transformation (function varianceStabilizingTransformation from package DESeq2 with parameter blind=TRUE).

- Hierarchical Clustering

Hierarchical clusterings of samples were performed using Ward’s agglomerative method, passing the euclidean distances between samples to function hclust from package stats (with parameter method="ward.D2"). Cluster stability was estimated by multiscale bootstrap resampling using function pvclust from package pvclust ^8^ with parameter nboot=1,000.

- Principal Component Analysis

Principal Component Analyses (PCA) of sample expression levels were performed with gene signals centered but not scaled (using function prcomp from package stats). PCA was performed based on the 1,000 genes with the highest variance in the experiment.

- Statistical Modeling

Starting from the filtered, non-normalized counts, the effective library sizes were computed using function calcNormFactors from the edgeR package ^8^. The mean-variance relationship was estimated using the voom function from the limma package ^9^ and injected into the statistical model as observational-level weights.

Linear regression models were applied using function lmFit from the limma package. Comparisons of interest were computed through statistical contrasts, using function makeContrasts from the limma package. The empirical Bayes method (function eBayes from the limma package) was used to compute moderated 𝑝-values. 𝑝-values were then corrected for multiple comparisons using Benjamini and Hochberg’s false discovery rate (here named adjusted p-value) controlling procedure (function p.adjust from package stats with parameter method="BH").

Genes were considered differentially expressed if they are significant (at adjusted 𝑝 < 0.05) and with fold-change > 2 or < 1/2. To compare DEGs in common between the comparison RS vs SS and RS vs SL, genes are considered positively correlated if they are significant (at adjusted 𝑝 < 0.05) and with fold-change > 2 or < 1/2 for both comparisons RS vs SS and RS vs SP. They are anti-correlated if they are significant (at adjusted 𝑝 < 0.05) for both effects and with fold-change > 2 for one effect and < 1/2 for the other (or vice-versa).

To illustrate overlaps in the three comparisons RS vs SS, RS vs SL, and SL vs SS, we draw a Venn diagram between DEGs (Fig. 2a and b).

**Gene Set Analyses**

- Gene annotation

Gene Ontology (GO) terms from the “Biological Process” tree were associated with genes with UniRef90 or Swiss-Prot annotations using the “ID mapping” data provided by UniProt ^10^.

- Competitive Gene Set Tests (MRGSE)

Gene set tests were applied to statistical contrasts using function geneSetTest ^11^ from the limma package (with parameters alternative="mixed", type="t" and ranks.only=TRUE). The method tests whether a set of genes is highly ranked relative to other genes in terms of their t-statistics (*competitive* approach). Only sets with at least 25 genes and at most 1000 were considered. Result tables (Table S3 and Table S4) report the 𝑝-values (column p.value), false discovery rate adjusted p.value (column Adjusted p.value), the number of genes in the gene set (column nb genes), and information for each identified gene set (columns db; id; description). To illustrate gene set overlaps between the three comparisons RS vs SS, RS vs SL, and SL vs SS, we draw a Venn diagram (using the top 50 terms of each three comparisons) (Fig. 3a).

1. Bunet, R. *et al.* First insight into the whole genome shotgun sequence of the endangered noble pen shell Pinna nobilis : a giant bivalve undergoing a mass mortality event. *Journal of Molluscan Studies* **87**, (2021).

2. López-Sanmartín, M. *et al.* Real-Time PCR based test for the early diagnosis of Haplosporidium pinnae affecting fan mussel Pinna nobilis. *PLOS ONE* **14**, e0212028 (2019).

3. MacManes, M. D. The Oyster River Protocol: a multi-assembler and kmer approach for de novo transcriptome assembly. *PeerJ* **6**, e5428 (2018).

4. Smith-Unna, R., Boursnell, C., Patro, R., Hibberd, J. M. & Kelly, S. TransRate: reference-free quality assessment of de novo transcriptome assemblies. *Genome Research* **26**, 1134–1144 (2016).

5. Seppey, M., Manni, M. & Zdobnov, E. M. BUSCO: Assessing Genome Assembly and Annotation Completeness. in *Methods in Molecular Biology* 227–245 (2019). doi:10.1007/978-1-4939-9173-0_14.

6. Soneson, C., Love, M. I. & Robinson, M. D. Differential analyses for RNA-seq: transcript-level estimates improve gene-level inferences. *F1000Research* **4**, 1521 (2016).

7. Anders, S. & Huber, W. Differential expression analysis for sequence count data. *Genome Biology* **11**, R106 (2010).

8. Robinson, M. D., McCarthy, D. J. & Smyth, G. K. edgeR: a Bioconductor package for differential expression analysis of digital gene expression data. *Bioinformatics* **26**, 139–140 (2010).

9. Smyth, G. K. Linear Models and Empirical Bayes Methods for Assessing Differential Expression in Microarray Experiments. *Statistical Applications in Genetics and Molecular Biology* **3**, 1–25 (2004).

10. Ashburner, M. *et al.* Gene Ontology: tool for the unification of biology. *Nature Genetics* **25**, 25–29 (2000).

11. Michaud, J. *et al.* Integrative analysis of RUNX1 downstream pathways and target genes. *BMC Genomics* **9**, 363 (2008).
